# Efficacy and safety testing of a COVID-19 era emergency ventilator in a healthy rabbit lung model

**DOI:** 10.1186/s42490-022-00059-x

**Published:** 2022-03-14

**Authors:** Luke A. White, Benjamin S. Maxey, Giovanni F. Solitro, Hidehiro Takei, Steven A. Conrad, J. Steven Alexander

**Affiliations:** 1 Department of Molecular & Cellular Physiology, LSU Health Shreveport, 1501 Kings Highway, Shreveport, LA 71103-3932 USA; 2 Department of Orthopedic Surgery, LSU Health Shreveport, Shreveport, LA USA; 3 Department of Pathology, LSU Health Shreveport, Shreveport, LA USA; 4 Department of Medicine, LSU Health Shreveport, Shreveport, LA USA; 5 Department of Emergency Medicine, LSU Health Shreveport, Shreveport, LA USA; 6 Department of Pediatrics, LSU Health Shreveport, Shreveport, LA USA; 7 Department of Neurology, LSU Health Shreveport, Shreveport, LA USA

**Keywords:** Ventilator, Emergency, Low-cost, COVID-19, Rabbit, Lung

## Abstract

**Background:**

The COVID-19 pandemic revealed a substantial and unmet need for low-cost, easily accessible mechanical ventilation strategies for use in medical resource-challenged areas. Internationally, several groups developed non-conventional COVID-19 era emergency ventilator strategies as a stopgap measure when conventional ventilators were unavailable. Here, we compared our FALCON emergency ventilator in a rabbit model and compared its safety and functionality to conventional mechanical ventilation.

**Methods:**

New Zealand white rabbits (*n* = 5) received mechanical ventilation from both the FALCON and a conventional mechanical ventilator (Engström Carestation™) for 1 h each. Airflow and pressure, blood O_2_ saturation, end tidal CO_2_, and arterial blood gas measurements were measured. Additionally, gross and histological lung samples were compared to spontaneously breathing rabbits (*n* = 3) to assess signs of ventilator induced lung injury.

**Results:**

All rabbits were successfully ventilated with the FALCON. At identical ventilator settings, tidal volumes, pressures, and respiratory rates were similar between both ventilators, but the inspiratory to expiratory ratio was lower using the FALCON. End tidal CO_2_ was significantly higher on the FALCON, and arterial blood gas measurements demonstrated lower arterial partial pressure of O_2_ at 30 min and higher arterial partial pressure of CO_2_ at 30 and 60 min using the FALCON. However, when ventilated at higher respiratory rates, we observed a stepwise decrease in end tidal CO_2_. Poincaré plot analysis demonstrated small but significant increases in short-term and long-term variation of peak inspiratory pressure generation from the FALCON. Wet to dry lung weight and lung injury scoring between the mechanically ventilated and spontaneously breathing rabbits were similar.

**Conclusions:**

Although conventional ventilators are always preferable outside of emergency use, the FALCON ventilator safely and effectively ventilated healthy rabbits without lung injury. Emergency ventilation using accessible and inexpensive strategies like the FALCON may be useful for communities with low access to medical resources and as a backup form of emergency ventilation.

**Supplementary Information:**

The online version contains supplementary material available at 10.1186/s42490-022-00059-x.

## Background

On March 11, 2020, worldwide infection with the novel SARS-CoV-2 virus/COVID-19 was declared a pandemic by the World Health Organization [1, 2]. Initial reports described severe respiratory disease, including acute respiratory distress syndrome (ARDS) that, in many cases, required ICU level care [3, 4]. Mechanical ventilation remains a pillar of supportive care for COVID-19 induced ARDS [5, 6]. There were concerns that demand for accessible and functional mechanical ventilators could easily exceed supply, and this would lead to extraordinarily difficult triage decisions by healthcare providers to prioritize resource allocation for patients [7, 8]. These issues persist and are too often encountered in low-and middle-income nations, where medical-grade equipment and supplies become inaccessible [9, 10].

For these reasons, many international scientific and clinical engineering groups began efforts to develop non-traditional, emergency-use mechanical ventilation approaches for use in medical resource-challenged settings [11, 12]. It was thought that such “COVID-19 era emergency ventilators” (CEEVs) could potentially be used on patients with mild to moderate cases of ARDS to allow for reallocation of more sophisticated ICU ventilators to patients with more severe ARDS, preventing the need to withdraw or refuse mechanical ventilation to a patient due to lack of supply [13]. Similar allocation strategies have been successfully employed using anesthetic gas machines in especially hard-hit regions, such as New York City and Northern Italy [14, 15]. Likewise, CEEVs could provide temporary but critical solutions when supplies of more sophisticated ventilators run short and must be restored through redistribution, an ordinarily lengthy process that was greatly exacerbated by pandemic-associated supply chain interruptions and import restrictions [16, 17].

Many designs for CEEVs have been advanced and publicized globally with assembly information and end-user instructions uploaded on websites [18–21]. Less commonly, these designs have undergone more rigorous testing, including safety and efficacy testing in preclinical animal models [22–27]. The lack of testing is likely due to resource and time constraints for performing such tests amidst a pandemic, and the urgency to disseminate the design in a timely manner.

We previously described and tested our own CEEV design called the FALCON [28]. The FALCON can be assembled from low-cost, off-the-shelf components. A unique characteristic of the FALCON is its ability to function immediately and predictably following assembly, without the need to initialize microcontroller programming. Despite its deliberately simple design, benchtop testing of the FALCON demonstrated its robustness in performing accurately and consistently. However, the efficacy and safety of the FALCON was not tested in vivo in the context of the complex physiology of an animal model.

In our present study, we tested the utility and safety of the FALCON in a healthy rabbit lung model and compared its function to an ICU rated mechanical ventilator. The crossover design of the study helped clarify subtle performance differences between these ventilators. As such, this study design could also be more broadly applied to other CEEVs and future novel ventilators to test their utility beyond benchtop testing in a more meaningful, clinical-like scenario.

## Materials and methods

The study was conducted with the approval of the Animal Care and Use Committee of LSU Health Shreveport (study protocol #S-21-001), and all animals and procedures were carried out in compliance with the Institution’s policies involving the care and use of laboratory animals. All methods are reported in accordance with ARRIVE guidelines. All experiments and sample collections were performed by the same technician.

### FALCON ventilator and ventilation circuit assembly

**Fig. 1:**
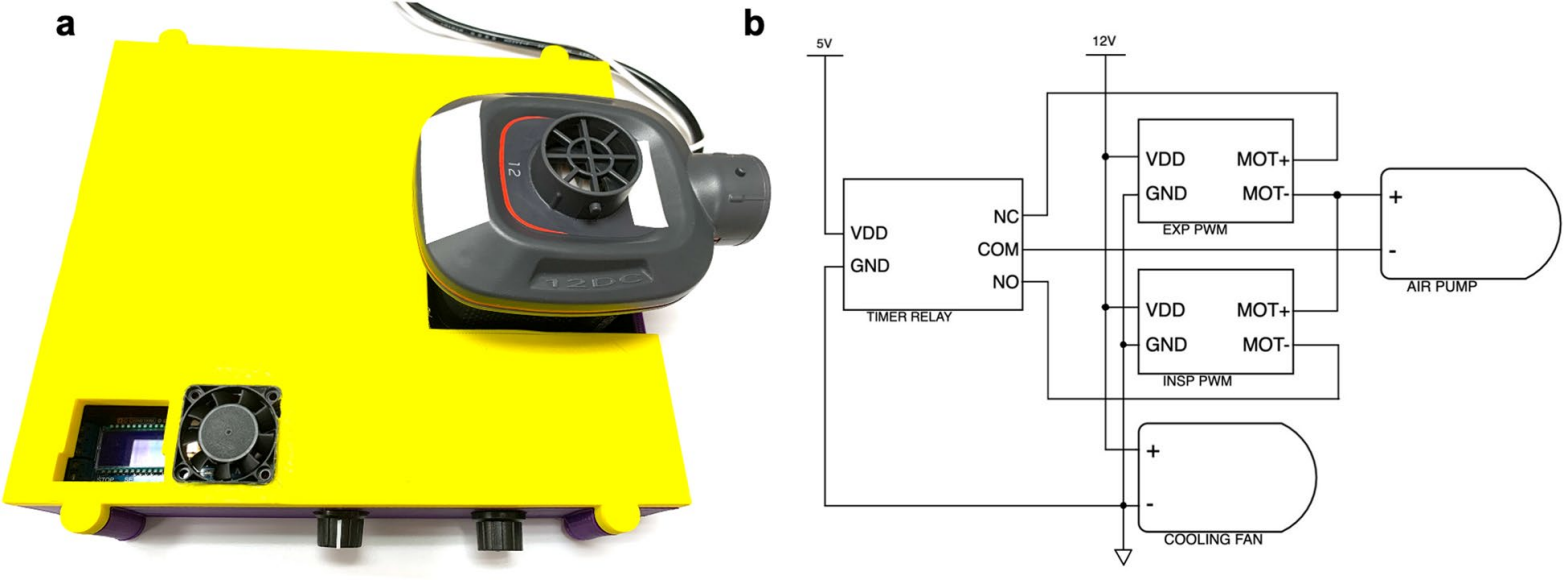
**a** Prototype of the FALCON ventilator, modified from the previously published design (see text for details). **b** Wiring schematic for the modified FALCON ventilator. 5 V/12 V five/twelve-volt power supply, COM common, EXP PWM expiratory pulse width modulator, GND ground, INSP PWM inspiratory pulse width modulator, MOT+/− positive/negative motor, NC normally closed, NO normally open, VDD voltage drain drain

The FALCON ventilator (Fig. 1a) was assembled as previously described [28], with a few notable modifications. The wiring between the air pump, timer relay, and pulse width modulators were modified (Fig. 1b) so that the current delivered to the fan was reversed during the expiratory phase. While the inspiratory pulse width modulator still controlled the peak inspiratory pressure (PIP), the expiratory pulse width modulator was used to actively slow down the turbine, rather than relying on the turbine to spin down passively, allowing for greater respiratory rates. With this wiring scheme change, the expiratory pulse width modulator lost the capability to control positive end expiratory pressure (PEEP), which was now set with a PEEP valve. This wiring scheme led to an increase in temperature experienced by the solenoid in the timer relay, potentially causing the solenoid to stall. To prevent overheating, a cooling fan (12 V DC brushless muffin fan, Hong Xing Shu Electronics Company LTD, Shenzhen, Guangdong, China) was fitted into the housing above the solenoid.

**Fig. 2:**
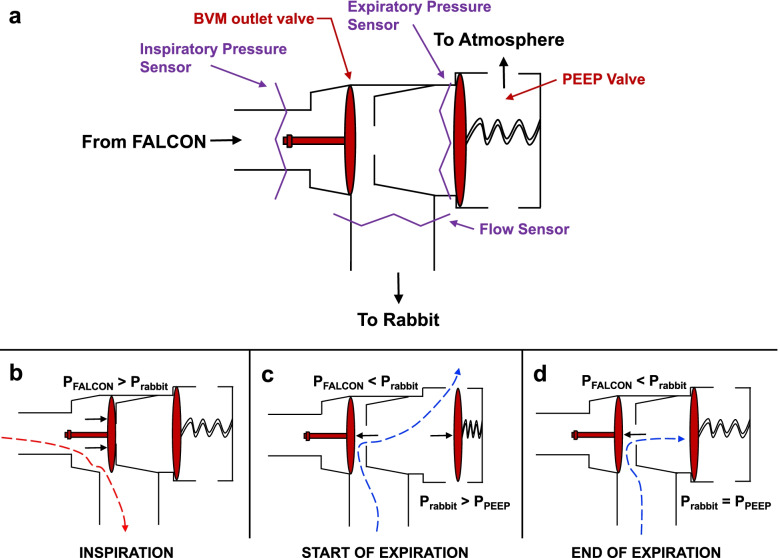
Schematic of the three-way, two-position pneumatically driven ventilator valve from the outlet side of an infant BVM used with the FALCON. **a** The FALCON was connected to the inspiratory side of the valve with a barometric pressure sensor to capture inspiratory pressure waveforms, and a PEEP valve was connected to the expiratory side with a second pressure sensor to capture expiratory pressure waveforms. The common line was used to connect the ventilator circuit to the rabbit, and a flow sensor was placed between the valve and rabbit to capture flow waveforms. **b** During inspiration, the FALCON’s air pump generated a positive pressure (P_FALCON_) which was greater than the airway pressure in the rabbit (P_rabbit_), causing the valve to open between the inspiratory side and the common and air to flow into the rabbit lungs. **c** During expiration, the turbine in the air pump rapidly slowed down, and P_FALCON_ rapidly dropped below the P_rabbit_, at which point the valve shut towards the inspiratory limb and opened to the expiratory outlet, allowing outflow of air from the rabbit lungs into atmosphere. **d** When P_rabbit_ reached the pressure set on the PEEP valve (P_PEEP_), the PEEP valve closed, and air ceased to flow out of the rabbit lungs

The ventilator circuit for the FALCON was assembled from conventional continuous positive airway pressure (CPAP) respiratory tubing and spare parts from a bag valve mask (BVM). The three-way, two-position pneumatically driven valve found on the outlet side of an infant BVM (SPUR II® infant model, AMBU® A/S, Columbia, MD, USA) was utilized to connect the FALCON to the rabbit and minimize the dead space. The FALCON was connected to the inspiratory side of the valve with silicone rubber fitted CPAP hosing (6 ft. × 19 mm inner diameter, Philips Respironics, Murrysville, PA), while a PEEP valve (Disposable PEEP Valve 20, AMBU® A/S, Columbia, MD, USA) was connected on the expiratory side. The common line was used to connect the ventilator circuit to the rabbit (Fig. 2a). During inspiration, the FALCON’s air pump generated a positive differential pressure gradient, causing the valve to open between the inspiratory side and the common (Fig. 2b). During expiration, the turbine in the air pump rapidly slowed down, and the pressure on the inspiratory limb was lower than the common. The valve then shut toward the inspiratory limb and opened to the expiratory outlet (Fig. 2c). Air flowed out the expiratory outlet and through the PEEP valve until the pressure in the common fell to the set PEEP (Fig. 2d).

### Animals and experimental design

The experiments were performed with healthy male New Zealand white rabbits (mechanical ventilation, or MV, group; *n* = 5), weighing between 2.0 to 4.0 kg. No exclusion criteria were set for the experiments, and all animals were included in the study.

**Fig. 3:**
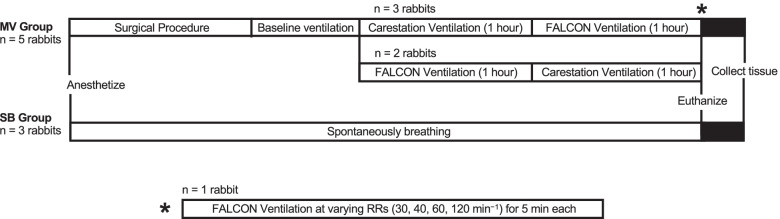
Experimental study design. Five rabbits underwent the experimental procedure (MV group). After anesthesia induction, the rabbits underwent the surgical procedure (see text for details) followed by baseline ventilation on the Carestation to determine ventilation settings for the remainder of the experiment. Afterwards, a subgroup of rabbits (*n* = 3) was ventilated with the Carestation first followed by the FALCON, the rest (*n* = 2) ventilated in the reverse order. One rabbit (indicated by *) was further ventilated on the FALCON at varying respiratory rates (at 30, 40, 60, and 120 breaths/min) for 5 min each. After ventilation, the rabbits were euthanized, and lung tissue samples were collected. Three spontaneously breathing rabbits (SB group) were anesthetized and euthanized for healthy lung tissue collection

In this crossover study, the rabbits received ventilation from both a hospital grade ventilator (Engström Carestation™, General Electric Healthcare, Chicago, IL) and the FALCON for 1 h each (Fig. 3). After determination of baseline ventilation settings on the Carestation, the rabbits were randomized (block randomization of 2 groups with 1 block size of 4 [29], final rabbit assigned a group via simple randomization from a coin flip) to receive ventilation either from the Carestation first followed by the FALCON (*n* = 3), or from the FALCON first followed by the Carestation (*n* = 2). Afterwards, the rabbits were euthanized, and samples of lung tissue were collected. One rabbit from the MV group underwent additional ventilation on the FALCON for approximately 20 min. Additionally, samples of lung tissue from spontaneously breathing healthy male New Zealand white rabbits (SB; *n* = 3) were taken to serve as a healthy control.

### Experimental protocol

**Fig. 4:**
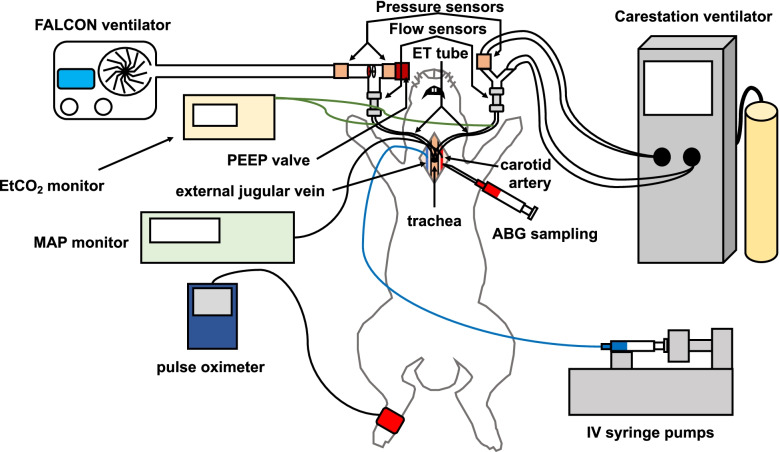
Experimental setup; see text for further details. ABG arterial blood gas, ET endotracheal, EtCO_2_ end tidal carbon dioxide, IV intravenous, MAP mean arterial pressure, PEEP positive end expiratory pressure

Anesthesia was induced with an intramuscular injection of xylazine (4 mg/kg) followed by a ketamine + acepromazine (40 mg/kg and 0.75 mg/kg, respectively) cocktail intramuscular injection, and the rabbit was placed in the supine position on a heating pad (see Fig. 4 for experimental setup). A rectal thermometer was inserted for core body temperature monitoring, and a pulse oximetry sensor was placed along a hind paw for continuous monitoring of oxygen saturation and pulse rate. A nosecone was placed and supplemental oxygen and isoflurane anesthetic (up to 5%, as needed) were administered. A midline incision along the ventral neck was made, and the external jugular vein was isolated and cannulated with a double-lumen venous catheter for continuous infusion of a ketamine + xylazine cocktail (10 mg/kg/hr. and 4 mg/kg/hr., respectively), and the isoflurane administration was stopped. Additionally, an intravenous infusion of fluids (5% dextrose with 0.9% saline) was begun and adjusted, when needed, for a total volume replacement of 4 mL/kg/hr. The common carotid was isolated and cannulated with a single lumen arterial catheter, and a pressure transducer was connected for continuous arterial blood pressure monitoring. Arterial blood was intermittently sampled at the arterial cannula for arterial blood gas (ABG) measurements. A horizontal incision was made in the trachea, and an endotracheal tube (3.0 mm inner diameter) was introduced and secured. The endotracheal tube was connected to the Carestation ventilator circuit, set in pressure control ventilation with the initial settings of PIP = 11 cm H_2_O, PEEP = 3 cm H_2_O, respiratory rate (RR) = 40 breaths/min, and inspiratory time to expiratory time (I:E) ratio = 1:1. A bolus of cisatracurium besylate (0.12 mg/kg) was intravenously administered to depress spontaneous breathing, and a continuous intravenous infusion (1.0 to 2.0 μg/kg/min) was then started to maintain cessation of spontaneous respiration. During baseline ventilation determination, the target PIP (PIP_target_) and PEEP (PEEP_target_) were determined by adjusting the PIP and PEEP to achieve a tidal volume (V_T_) < 10 mL/kg, blood oxygen saturation (SpO_2_) > 95%, and end tidal carbon dioxide (EtCO_2_) between 35 and 45 cm H_2_O for at least 10 min. These ventilation settings remained constant throughout the remainder of each experiment, as this provided a means to directly compare the ventilator performance between the Carestation and FALCON, given identical ventilation settings. The rabbit was mechanically ventilated for 1 h each on the Carestation and FALCON, and the order of ventilation was randomly assigned to each rabbit prior to the experiment. After ventilation with both ventilators, the rabbit was euthanized with intravenous administration of pentobarbital (100 mg/kg).

In this experimental protocol, baseline ventilation with the Carestation was used to set the ventilation settings for both the Carestation and FALCON for the rest of the experiment, meaning that the settings may not be optimal for the FALCON. Although the FALCON may perform differently than the Carestation at identical ventilation settings, an alteration to those settings (e.g., the respiratory rate, or RR), given certain constraints to PIP and PEEP may allow for more appropriate ventilation with the FALCON. To test this, one rabbit from the MV group was ventilated with the FALCON at varying RRs (30, 40, 60, and 120 breaths/min) for 5 min each with constant pressure settings (PIP_target_ = 11 cm H_2_O, PEEP_target_ = 3 cm H_2_O). This was performed as an additional step after ventilating with the Carestation and FALCON for 1 h each.

In the SB rabbit group (*n* = 3), anesthesia was induced with a xylazine (4 mg/kg) intramuscular injection followed by a ketamine + acepromazine (40 mg/kg and 0.75 mg/kg, respectively) cocktail intramuscular injection. A nosecone was placed, and supplemental oxygen and isoflurane (5%) were administered. A marginal ear vein catheter was inserted, and intravenous pentobarbital (100 mg/kg) was administered for euthanasia.

### Physiological and respiratory mechanics measurements

Invasive arterial pressure was continuously measured at the arterial cannulation site with pressure transducers (Cobe Laboratories, McHenry, Illinois, United States) connected to a blood pressure monitor (Pressure Monitor BP-1, World Precision Instruments, Sarasota, Florida, United States). The blood pressure monitor was connected to a data acquisition unit (PowerLab 4/30, AD Instruments, Colorado Springs, Colorado, United States) to transfer readings to a computer (Optiplex 7070, Dell Inc., Round Rock, TX) for recording with the manufacturer’s software (Labchart 7, AD Instruments, Colorado Springs, Colorado, United States). Mean arterial pressure (MAP) was determined using the recorded traces with the manufacturer’s software. SpO_2_ and pulse rate were continuously measured with a veterinary pulse oximeter (Contec CMS60D-VET pulse oximeter, Contec Medical Systems, Qinhuangdao, China) and recorded to the computer with the manufacturer’s software. EtCO_2_ and core body temperature were continuously measured with a physiological monitoring system (CapnoScan, Kent Scientific, Torrington, Connecticut, United States), and data were exported digitally to the computer following the manufacturer’s instructions.

Airflow measurements were taken at the common line in the ventilator circuits utilizing a proximal airflow sensor (SFM3400-D, Sensirion AG, Staefa, Switzerland), which had a dead space volume less than 1 mL. Data from the sensor were transferred to the computer with a USB sensor cable (Evaluation Kit EK-F3x-CAP, Sensirion AG, Staefa, Switzerland) and accompanying software provided by the manufacturer. To record pressure waveforms, two barometric pressure sensors (DPS310, Infineon Technologies AG, Neubiberg, Germany) placed and sealed in custom stereolithography 3D printed housing (surgical guide resin printed with the Formlabs Form 2 printer, Somerville, Massachusetts, United States). Unlike the flow sensor, the pressure sensors had considerable dead-space (greater than 10 mL), so two pressure sensors were used with the FALCON ventilator circuit: one on the inspiratory line just before the 3-way, 2-position valve to record pressures during inspiration, and one on the expiratory side, just before the PEEP valve to record pressures during expiration. Only one pressure sensor was used with the Carestation ventilator circuit, just prior to the wye-piece on the inspiratory line, as the inspiratory and expiratory lines were not isolated from each other by a valve. Data from pressure sensors were sent to the computer via USB, following the instructions provided by the manufacturer. Using custom code written in Python (version 3.7.2, Python Software Foundation, Beaverton, Oregon, United States; all Python code available in the supplementary material), pressure readings captured by the two pressure sensors from the FALCON were combined to generate a single pressure waveform. Additionally, custom code was written in Python to determine V_T_, RR, I:E ratio, PIP, and PEEP for each respiratory cycle from the recorded flow and pressure waveforms. V_T_s were calculated from the areas under the flow waveform during inspiration. RRs and I:E ratios were determined from analysis of the flow waveform. PIPs and PEEPs were determined from the recorded pressure waveforms, and difference in PIP and PEEP from PIP_target_ and PEEP_target_ were calculated as ΔPIP = PIP − PIP_target_ and ΔPEEP = PEEP − PEEP_target_, respectively.

### Blood gas analysis

A blood gas analyzer (Radiometer ABL800 Flex, Radiometer Medical, Bronshoj, Denmark) was used to determine arterial partial pressure of oxygen (PaO_2_), arterial partial pressure of carbon dioxide (PaCO_2_), pH, and lactate concentrations from arterial blood samples taken after 30 and 60 min of ventilation with both the Carestation and FALCON.

### Tissue fixation and processing

Lung tissue was fixed in 4% formaldehyde for 24 h using the tracheal ligation technique [30]. Briefly, the endotracheal tube was removed, and the trachea was ligated with 4–0 suture below the tracheal incision site to keep the lungs inflated. A thoracotomy was carefully performed, the lungs were inspected for pneumothorax. The heart and lungs were removed *en bloc*, ensuring the lungs remained inflated. The right main bronchus was also ligated with 4–0 suture to prevent lung deflation, and a sample of the right anterior lobe was excised for wet to dry weight measurements. A weight was then tied to the trachea, lungs, and heart unit, and all were submerged in 4% formaldehyde for 24 h.

### Lung wet weight to dry weight measurements

Samples of lung tissue from the right anterior lobe were excised, and weight measurements were taken before and after drying at 47 °C for 96 h. The wet to dry weight ratio was calculated as the wet weight divided by the dry weight.

### Lung histology

After fixation, a section of the middle portion of the left posterior lobe was excised and processed prior to embedding in paraffin, following the institution’s standard protocol. Tissue sections (10 μm) were stained with hematoxylin and eosin (H&E). A pathologist blinded to group allocations performed ventilator induced lung injury (VILI) scoring, as previously described [31], on three consecutive sections. Four metrics (alveolar congestion, hemorrhage, leukocyte infiltration, and thickness of alveolar wall) were scored on a 0–4 scale, where 0 represented normal lung, 1 mild (less than 25%) lung involvement, 2 moderate (25 to 50%) lung involvement, 3 severe (50 to 75%) lung involvement, and 4 very severe (75 to 100%) lung involvement, and an overall score was calculated as the average of all four metrics.

### Poincaré plot analysis

Poincaré plot analysis can be used to evaluate the variation of data in a time-series [32]. In the Poincaré plot, a datapoint at time *n* is plotted in the abscissa against its subsequent datapoint at time *n + 1* along the ordinate. The standard deviation of this dataset perpendicular to the line of identity *n = n + 1* is defined as SD1 and is a measure of variation from one datapoint to the immediate subsequent datapoint (short-term variation), while the standard deviation parallel to the line of identity (SD2) is a measure of all other variation (long-term variation). Poincaré plots were constructed for the PIP and PEEP generated by the Carestation and FALCON for each rabbit for analysis of short- and long-term variation in pressure generation.

### Statistical analysis

Data are presented as mean ± standard deviation, unless otherwise noted. For paired data, the Shapiro-Wilk test was performed to assess normality. For the data normally distributed, differences between the groups were evaluated with a two-tailed paired Students *t*-test. If the data were not normally distributed, the two-tailed Wilcoxon signed rank test was performed instead. Sample size for the crossover ventilation experiments (*n* = 5) was determined using an online sample size calculator (power = 0.8, α = 0.05) assuming that a physiologically significant difference in SpO_2_ between the FALCON and Carestation would occur if the mean paired differences were 10% with a standard deviation of paired differences of 5% [33]. For unpaired data, the two-tailed Mann-Whitney *U* test was performed. A *p* value < 0.05 was considered statistically significant. Besides the sample size determination, all statistics were performed with the Graphpad prism statistics software (version 9.2, Graphpad, San Diego, CA).

## Results

### The FALCON generated V_T_, PIP and PEEP comparably to the Carestation but spent less time in inspiration

**Fig. 5:**
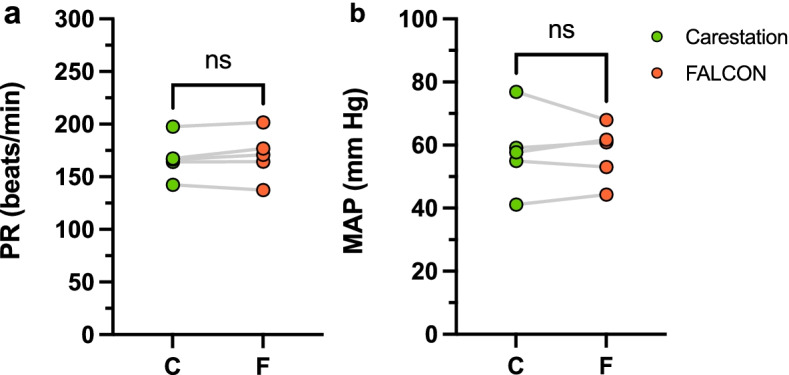
PR (**a**) and MAP (**b**) during ventilation with the Carestation (green) and FALCON (orange, *n* = 5 rabbits, two-tailed paired Students *t*-test, α = 0.05). MAP mean arterial pressure, NS not significant, PR pulse rate

Five (*n* = 5) rabbits in the experimental group were alternatively ventilated with the FALCON and Carestation for 1 h each. All five rabbits survived to the end of the trial and demonstrated similar mean arterial blood pressures (58 ± 13 mmHg Carestation, 58 ± 9 mmHg FALCON, *p* = 0.87) and pulse rates (168 ± 20 beats/min Carestation, 170 ± 23 beats/min FALCON, *p* = 0.33) for the duration of the experiment (Fig. 5).

**Fig. 6:**
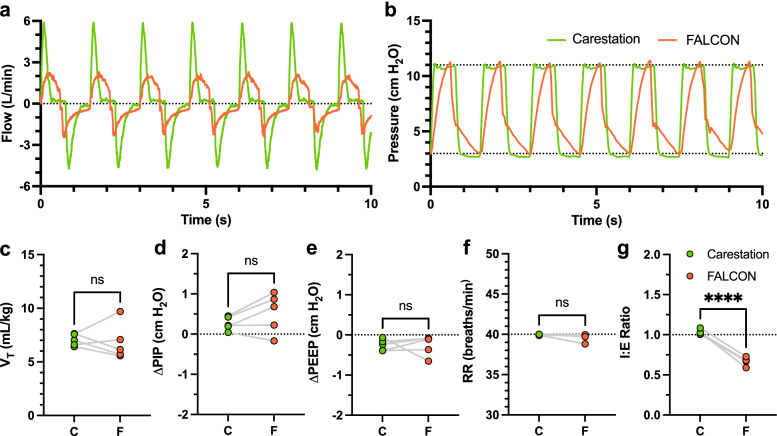
Representative flow (**a**) and pressure (**b**) waveforms generated by the Carestation (green line) and FALCON (orange) sampled over 10 s. V_T_ (**c**), ΔPIP (**d**), ΔPEEP (**e**), RR (**f**), and I:E ratio (**g**) derived from the average of 1-min samples taken every 10 min for the duration of ventilation are shown for the Carestation (green) and FALCON (orange). ΔPIP calculated as ΔPIP = PIP − PIP_target_, and ΔPEEP calculated as ΔPEEP = PEEP − PEEP_target_. Either two-tailed paired Students *t*-test (V_T_, ΔPIP, ΔPEEP, and I:E ratio) or Wilcoxon signed rank test (RR) were performed (*n* = 5 rabbits, α = 0.05). ****p < 0.0001, I:E inspiratory time to expiratory time, NS not significant, PEEP positive end expiratory pressure, PIP peak inspiratory pressure, RR respiratory rate, V_T_ tidal volume

Under similar conditions, compared to the Carestation, the flow waveform of the FALCON peaked at lower values and were more elongated (Fig. 6a), demonstrating that under similar settings, V_T_ was achieved less quickly with the FALCON compared to the Carestation. Additionally, pressure waveforms for the Carestation were more square-like, while the waveforms generated by the FALCON were more sawtooth (Fig. 6b), indicating that PIP and PEEP were reached later in the inspiratory and expiratory phases.

One-minute samples of the flow and pressure waveforms, taken at the start of the 1-h ventilation period and again every 10 min thereafter for both FALCON and Carestation, were analyzed to determine the average V_T_, ΔPIP, ΔPEEP, RR and I:E ratios for each rabbit during the ventilation period (Fig. 6c-g). Average V_T_s (7.1 ± 0.6 mL/kg Carestation, 6.8 ± 1.7 mL/kg FALCON, *p* = 0.77), ΔPIPs (0.3 ± 0.2 cm H_2_O Carestation, 0.5 ± 0.5 cm H_2_O FALCON, *p* = 0.16), ΔPEEPs (− 0.2 ± 0.1 cm H_2_O Carestation, − 0.3 ± 0.2 cm H_2_O FALCON, *p* = 0.95) and RRs (40.0 ± 0.1 breaths/min Carestation, 39.7 ± 0.5 breaths/min FALCON, *p* = 0.19) were not significantly different between ventilation with the FALCON versus the Carestation. However, the I:E ratios generated by the FALCON were significantly lower compared to the Carestation (1.03 ± 0.03 Carestation, 0.67 ± 0.05 FALCON, ****p < 0.0001), despite the timer relay on the FALCON being set at a 1:1 ratio. This appeared to indicate that using comparable settings, less time was spent in inspiration and more time in expiration with the FALCON for each respiratory cycle.

### At identical settings, oxygen and carbon dioxide gas exchange occurred less with the FALCON compared to the Carestation

At identical settings, less time was spent in inspiration with the FALCON versus the Carestation, and V_T_ was achieved less quickly. This may have caused a lower rate of gas exchange to occur with the FALCON. ABGs taken at 30 and 60 min (Table 1) demonstrated a moderately lower but significant decrease in PaO_2_ at 30 min with the FALCON (77.0 ± 9.9 mmHg) versus the Carestation (90.7 ± 18.6 mmHg, *p < 0.05). Despite this decrease, arterial saturation could be adequately maintained. PaCO_2_ levels were elevated at both the 30-min (33.4 ± 3.4 mmHg Carestation, 45.2 ± 5.5 mmHg FALCON, **p < 0.01) and 60-min (32.1 ± 4.1 mmHg Carestation, 45.1 ± 7.7 mmHg FALCON, **p < 0.01) timepoints with the FALCON versus the Carestation, and this led to a less alkalotic pH at both timepoints (at 30 min, 7.572 ± 0.069 Carestation, 7.461 ± 0.074 FALCON, **p < 0.01; at 60 min, 7.564 ± 0.061 Carestation, 7.450 ± 0.112 FALCON, **p < 0.01). Additionally, blood lactate levels were not different between the FALCON and Carestation groups (at 30 min, 2.7 ± 1.4 mmol/L Carestation, 2.4 ± 1.3 mmol/L FALCON, *p* = 0.29; at 60 min, 2.9 ± 1.4 mmol/L Carestation, 2.7 ± 1.5 mmol/L FALCON, *p* = 0.78).

**Table 1:** Arterial blood gas measurements

ABG Measurement	30 min			60 min		
	Carestation (*n* = 5)	FALCON (*n* = 5)	*p* value^†^	Carestation (*n* = 4)	FALCON (*n* = 5)	*p* value^‡^
PaO_2_ (mm Hg)	90.7 ± 18.6	77.0 ± 9.9	*	96.8 ± 17.7	80.8 ± 14.9	0.38
PaCO_2_ (mm Hg)	33.4 ± 3.4	45.2 ± 5.5	**	32.1 ± 4.1	45.1 ± 7.7	**
pH	7.572 ± 0.069	7.461 ± 0.074	**	7.564 ± 0.061	7.450 ± 0.112	**
lactate (mmol/L)	2.7 ± 1.4	2.4 ± 1.3	0.29	2.9 ± 1.4	2.7 ± 1.5	0.78

Data are presented with mean ± standard deviation

*ABG* Arterial blood gas, *PaCO*_*2*_ Arterial partial pressure of carbon dioxide, *PaO*_*2*_ Arterial partial pressure of oxygen

† Reported *p* value from two-tailed paired Students *t*-test (*n* = 5 rabbits, α = 0.05) of the ABG measurements from 30 min of ventilation with either the Carestation or FALCON

‡ Reported *p* value from either the paired Students *t*-test (PaCO_2_, pH and lactate) or Wilcoxon signed-rank test (PaO_2_) of ABG measurements from 60 min of ventilation with either the Carestation (*n* = 4 rabbits due to unanticipated inaccessibility of the ABG machine for one rabbit’s sample) or FALCON (*n* = 5 rabbits, α = 0.05, unpaired data point was ignored in the calculation)

*p < 0.05, **p < 0.01

**Fig. 7:**
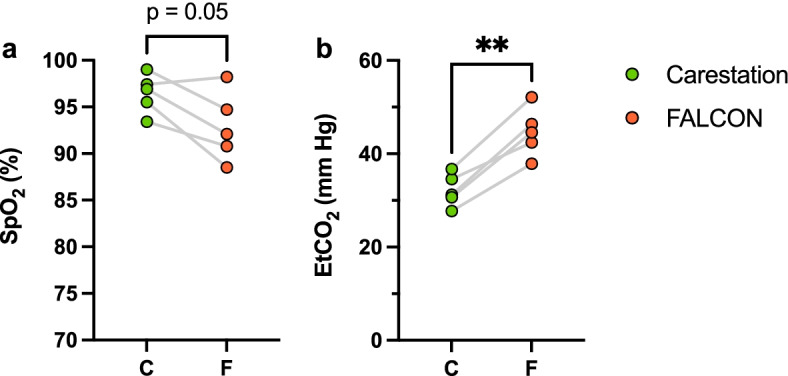
Average SpO_2_ (**a**) and EtCO_2_ (**b**) during ventilation with the Carestation and FALCON (*n* = 5 rabbits, two-tailed paired Students *t*-test, α = 0.05). **p < 0.01, EtCO_2_ end tidal carbon dioxide, SpO_2_ blood oxygen saturation

The average SpO_2_ over the course of ventilation for the FALCON trended lower compared to the Carestation (Fig. 7a; 96% ± 2% Carestation, 93% ± 4% FALCON, *p* = 0.05). Furthermore, the average EtCO_2_ was greater for the FALCON than the Carestation (Fig. 7b; 32 ± 4 mmHg Carestation, 45 ± 5 mmHg FALCON, **p < 0.01).

### By adjusting the respiratory rate, the FALCON achieved a broad range of adequate minute ventilation rates at given target PIP and PEEP

One rabbit was ventilated with the FALCON for 5 min at varying target RRs (30, 40, 60, and 120 breaths/min) to assess the ability of the FALCON to provide different minute ventilations (minute V̇), given specific constraints on PIP and PEEP. The FALCON was capable of cycling between inspiration and expiration at the target RRs while still achieving PIP_target_ and PEEP_target_ throughout the 5-min ventilation period (Table 2; maximal average ΔPIP = 1.7 ± 0.3 cm H_2_O at target RR = 60 breaths/min; maximal average ΔPEEP = 0.9 ± 0.1 cm H_2_O at target RR = 120 breaths/min). The average V_T_ over the 5-min ventilation period remained above 5 mL/kg for RR = 30, 40, and 60 breaths/min, although this fell to 2.9 mL/kg at RR = 120 breaths/min. The average SpO_2_, when sampled from the last minute of ventilation, remained above 97% at all respiratory rates.

**Table 2:** FALCON respiratory mechanics and SpO_2_ at different respiratory rates

Target RR (breaths/min)	Measured RR (breaths/min)	V_T_ (mL/kg)	ΔPIP (cm H_2_O)	ΔPEEP (cm H_2_O)	SpO_2_ (%)
30	29.8 ± 2.0	5.8 ± 0.4	1.0 ± 0.4	−0.4 ± 0.4	98
40	39.8 ± 0.2	5.7 ± 0.5	0.1 ± 0.1	−0.7 ± 0.6	99
60	59.4 ± 3.5	5.5 ± 0.4	1.7 ± 0.3	0.0 ± 0.3	98
120	119.6 ± 2.4	2.9 ± 0.3	0.6 ± 0.5	0.9 ± 0.1	99

Respiratory mechanics measured on one rabbit while ventilating at varying target RRs (30, 40, 60, and 120 breaths/min) and maintaining constant pressure settings (PIP_target_ = 11 cm H_2_O; PEEP_target_ = 3 cm H_2_O) for 5 min each. Measured RR, VT, ΔPIP, and ΔPEEP are presented as mean ± standard deviation from the full duration of the 5-min ventilation; SpO_2_ presented as mean SpO_2_ during the last minute of the 5-min ventilation. ΔPIP calculated as ΔPIP = PIP – PIP_target_, and ΔPEEP calculated as ΔPEEP = PEEP – PEEP_target_

*PEEP* Positive end expiratory pressure, *PIP* Peak inspiratory pressure, *RR* Respiratory rate, *SpO*_*2*_ Blood oxygen saturation, *V*_*T*_ Tidal volume

**Fig. 8:**
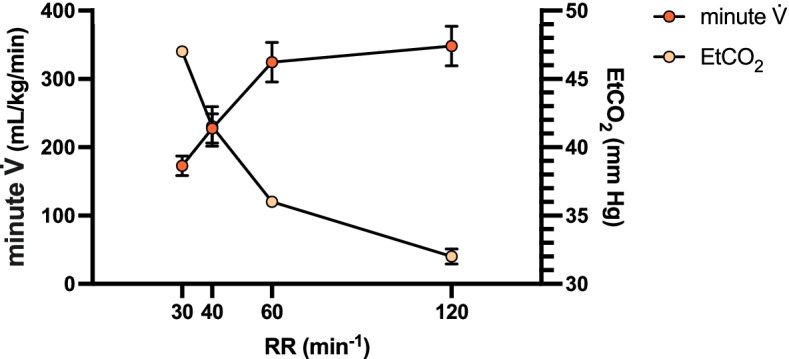
Results of minute V̇ and EtCO_2_ of a rabbit (*n* = 1) during mechanical ventilation with the FALCON for 5 min at varying target RRs (30, 40, 60, and 120 breaths/min) with constant pressure settings (PIP_target_ = 11 cm H_2_O; PEEP_target_ = 3 cm H_2_O). Minute V̇ presented is the mean minute V̇ from each full 5-min period. EtCO_2_ presented is the mean EtCO_2_ for the last minute of each 5-min ventilation period. Error bars, where present, indicate standard deviation. EtCO_2_ end tidal carbon dioxide, PEEP_target_ target positive end expiratory pressure, PIP_target_ target peak inspiratory pressure, RR respiratory rate, V̇ ventilation

Furthermore, when the RR was increased from 30 to 120 breaths/min, the average minute V̇, when sampled from the full 5 min, doubled from 173 ± 1 mL/kg/min to 348 ± 3 mL/kg/min (Fig. 8). This led to a decrease in the average EtCO_2_, sampled from the last minute of ventilation, from 47 ± 0 mmHg to 32 ± 1 mmHg.

### The FALCON PIP had greater short-term and long-term variability compared to the Carestation

**Fig. 9:**
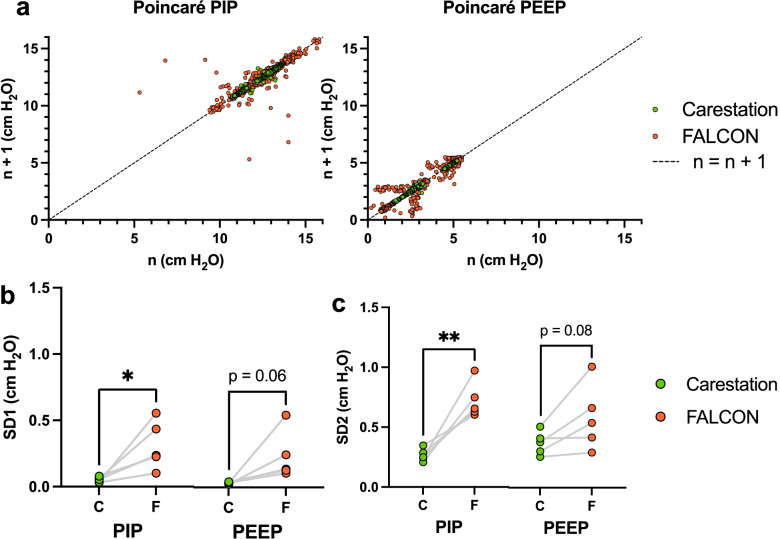
**a** Poincaré plots of PIP and PEEP with both the FALCON and Carestation compiled from all rabbits ventilated (*n* = 5 rabbits). Dotted line indicates the line of identity (*n = n + 1*). Average SD1 (**b**) and SD2 (**c**) for each rabbit ventilated with the FALCON and Carestation. Either a two-tailed paired Students *t*-test (SD1 PIP, SD2 PIP, and SD2 PEEP) or Wilcoxon signed rank test (SD1 PEEP) were performed (*n* = 5 rabbits, α = 0.05). *p < 0.05, **p < 0.01, PEEP positive end expiratory pressure, PIP peak inspiratory pressure

Poincaré plots of the PIP and PEEP produced by the FALCON and Carestation were generated for each rabbit (a compiled Poincaré plot from all rabbits is shown in Fig. 9a), and the short-term (SD1) and long-term (SD2) variations were calculated for each rabbit (Fig. 9b and c). The FALCON generated significantly higher short-term and long-term variation in PIP compared to the Carestation, although the variation remained less than 1 cm H_2_O (for SD1, 0.052 ± 0.02 cm H_2_O Carestation, 0.31 ± 0.18 cm H_2_O FALCON, *p < 0.05; for SD2, 0.27 ± 0.05 cm H_2_O Carestation, 0.72 ± 0.15 cm H_2_O FALCON, **p < 0.01). The short-term and long-term variation in PEEP generated by the FALCON were also less than 1 cm H_2_O and trended higher than the PEEP from the Carestation (for SD1, 0.03 ± 0.01 cm H_2_O Carestation, 0.23 ± 0.18 cm H_2_O FALCON, *p* = 0.06; for SD2, 0.37 ± 0.10 cm H_2_O Carestation, 0.58 ± 0.27 cm H_2_O FALCON, *p* = 0.08).

### Mechanical ventilation from the FALCON and Carestation did not lead to VILI during short term ventilation

**Fig. 10:**
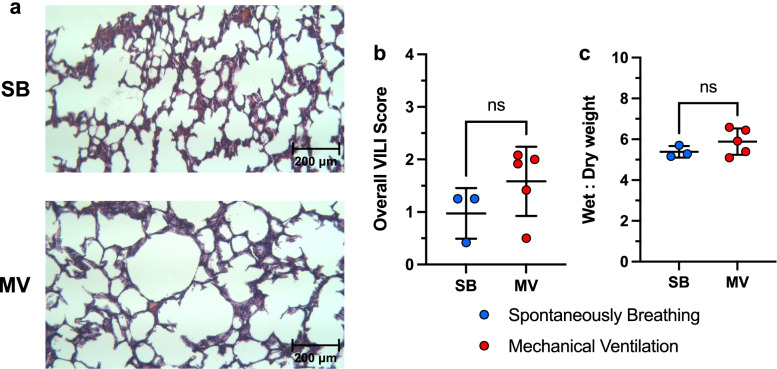
**a** Representative H&E staining (100x). **b** VILI overall histology score from lung samples (right posterior lobe) of spontaneously breathing rabbits (SB, *n* = 3) and rabbits mechanically ventilated (MV, *n* = 5) with both the Carestation and FALCON (two-tailed Mann-Whitney *U* test, α = 0.05). **c** Lung wet weight to dry weight ratio (two-tailed Mann-Whitney *U* test, α = 0.05). H&E hematoxylin and eosin, MV mechanical ventilation, NS not significant, SB spontaneously breathing

To assess the safety of ventilation with the FALCON, samples of lung tissue were taken after ventilation on both the Carestation and FALCON. Upon inspection after thoracotomy, no lungs appeared collapsed. Samples were then fixed and processed for H&E staining (Fig. 10a). A blinded pathologist examined the samples and scored for VILI (Fig. 10b), which demonstrated no significant difference between the MV and SB rabbits (1.0 ± 0.5 SB, 1.6 ± 0.7 MV, *p* = 0.13). Additionally, wet to dry weight ratios of the lung tissues (Fig. 10c) showed no significant difference between the MV and SB groups (5.4 ± 0.3 SB, 5.9 ± 0.6 MV, *p* = 0.39).

## Discussion

In the present study, we compared ventilation with the FALCON and Carestation ventilators in a healthy rabbit lung model. We found that V_T_, PIP, PEEP, and RR were comparable between the two ventilators. This validated our previous benchtop study that showed similarities between these approaches [28]. However, the present study showed that the measured I:E ratio on the FALCON was lower than the set value on the FALCON. This reflects delays in time spent by the turbine (1) accelerating from expiration to inspiration and (2) decelerating from inspiration to expiration. Consequentially, V_T_ was achieved more slowly, and the lungs remained fully expanded for a shorter time, possibly leading to the decreased oxygen uptake and carbon dioxide removal with the FALCON compared to the Carestation set at identical settings. We also showed that the FALCON can produce RRs (up to 120 breaths/min) much greater than that utilized in the crossover protocol (40 breaths/min) with a subsequent increase in minute ventilation, indicating that the settings can be easily altered to achieve favorable gas exchange.

The prolonged flow and pressure waveforms generated by the FALCON had been observed in other CEEVs [23, 27]. Crossover animal studies like ours in other CEEVs may reveal subtle, yet similar, differences between these and conventional ventilators. Such direct comparisons are valuable as they demonstrate how clinicians may need to adjust settings used on CEEVs to perform with similar efficacy to conventional ventilators.

On the Poincaré plot analysis, the FALCON demonstrated slightly higher short-term and long-term variations in PIP compared to the Carestation. This was likely due to the differences on how the PIP was generated. The Carestation generated pressures by regulating a high pressure gas source, such as a tank of oxygen or normal air, with electronically-driven pressure regulators and stepping the pressure down to the set pressure values [34], leading to highly reproducible PIP and PEEP. With the FALCON, PIP was generated by an accelerating air turbine. Even slight disparities in current delivered to the turbine may have led to differences in air speed produced by the turbine, resulting in the observed variations seen in PIP between one respiratory cycle and the next. In contrast, the PEEP in the FALCON was generated with a PEEP valve, which is a spring-loaded valve whose closing pressure was set independently of the FALCON’s electronics [35]. This is likely why lower variations were observed with the FALCON’s PEEP compared to its PIP, and why short-term and long-term variation was only trending higher with the FALCON’s PEEP compared to the PEEP produced by the Carestation. Regardless, the short- and long-term variation of PIP and PEEP seen with the FALCON would not likely be noticeably different than the Carestation clinically, as both remained less than 1 cm H_2_O.

There were several limitations to the current study. First, although there were no differences in wet to dry weight ratios or VILI scoring between the MV and SB groups, the relatively short ventilation time of 1 h with each ventilator is not long enough to fully assess the longer-term efficacy and safety profile of the FALCON. Many triggers of VILI—including barrier dysfunction [36], local pro-inflammatory pathway activation and leukocyte recruitment [37, 38], and oxidative stress [39]—occur secondary to the initial injury of mechanical trauma and hyperoxia [40–43], evolve over the course of hours to days, and can lead to multisystem organ injury and even failure, a process termed biotrauma [40, 44]. Nevertheless, any significant histological evidence of VILI was not observed over this short time-course. This study provides an initial proof-of-concept that the FALCON can be used as a mechanical ventilator without immediate devastating effects, such as pneumothorax, and future studies would include longer ventilation times.

Another limitation of our study is the animal model that we selected. We chose to perform these initial experiments in a healthy lung rabbit model, as failure to adequately ventilate in healthy lungs would certainly mean failure in an ARDS model. This allowed us to look for the presence of VILI without confounding evidence of lung injury incurred intentionally from a disease model, but it was not confirmed whether the FALCON can adequately ventilate injured lungs. Furthermore, studies on larger animals, such as pigs, would better model adult human pulmonary physiology and would validate that the FALCON can achieve appropriate PIPs and PEEPs at larger V_T_s. Future studies would involve the testing of the FALCON in a larger animal model with varying degrees of ARDS severity [45].

A third limitation of the experimental design was the titration of PIP and PEEP based on measured V_T_ prior to ventilation with the FALCON, which is not necessarily reflective of how the FALCON would be used in a clinical scenario. The FALCON lacks a flow sensor and thus lacks V_T_ monitoring. Decisions on pressure setting and adjustment would likely then be made without knowing V_T_, limiting the clinician’s capacity to properly select appropriate pressures to achieve adequate ventilation without potentially inducing injury.

The FALCON has some strengths over some of its CEEV counterparts, many of which—along with the FALCON’s considerable limitations—are discussed in our previous benchtop study [28]. In particular, the FALCON does not require the use of a microcontroller and subsequent programming, meaning that very little knowledge of electronics is needed to properly assemble and implement its use. The FALCON’s use of a turbine for pressure generation means that it does not require pressurized gas hook-ups to operate, although electrical power is required. Furthermore, the FALCON can be assembled cheaply and quickly from widely available low-cost, off-the-shelf components.

However, like all CEEVs, the FALCON also has considerable limitations compared to its commercial ventilator counterparts. In particular, the FALCON has no direct control of the fraction of inspired oxygen above room air. Additionally, the FALCON has no built-in alarms to alert the user of adverse events, such as overpressure, inadequate or too high V_T_ delivery, patient disconnect or apnea, turbine or power failure, and presence of air leaks. The FALCON does not contain electronic pressure or flow sensors to ensure either target PIP, PEEP, or V_T_ are achieved through feedback control, although a pressure manometer is present for manual pressure monitoring. This was done deliberately to maintain the simplicity of the FALCON, even though such feedback implementation could potentially have prevented the differences between the target and measured I:E ratio that was observed in this study. Furthermore, feedback control could also ensure target respiratory parameters are met during dynamic changes of a patient’s pulmonary status, including pulmonary compliance and airway resistance, a common occurrence during a patient’s disease progression. CEEVs span a wide range of sensor and alarm implementation, and others have also opted to not implemented alarms or sensors [26, 27]. However, the use of modular pressure and flow sensors and alarms could reduce the incidence of complications that arise from the lack of built-in ones [46, 47].

The rapid spread of SARS-CoV-2 virus infection and its variants has tested the global medical infrastructure. Life-saving tools, including mechanical ventilators, typically require extensive development, testing and manufacturing, all of which are timely and costly. Consequently, the normal supply of ventilators is typically kept relatively low, but this can create devastating shortages during critical surges. Development of impromptu medical devices, such as CEEVs, has exploded since the start of the pandemic, providing a valuable substitute in case of supply shortages [11, 12]. Further studies into their efficacy and safety, such as the one presented here, and adherence to guidelines set forth by regulatory agencies, such as the US Food and Drug Administration [48] and the UK Medicines and Healthcare Product Regulatory Agency [49], can allow for development of emergency mechanical ventilator designs that can be rapidly, effectively, and more safely deployed in future surge crises, particularly in medical resource challenged areas and in response to advanced diagnostic approaches [50–52]. Additionally, our crossover study design in an animal model helped elucidate differences between a CEEV and conventional mechanical ventilator—for example, a difference in EtCO_2_—that may not be detected with benchtop testing.

Finally, it should be noted that the FALCON is not approved for human use under the United States Food and Drug Administration (FDA) or with any other regulatory agency and should not be used as a lifesaving therapy without further validation and approval.

## Conclusion

The FALCON can successfully ventilate lungs in a healthy anesthetized rabbit model. Further studies are needed to determine the FALCON’s potential use in ARDS. Although conventional mechanical ventilators are clearly preferable, our study demonstrates how CEEVs and future emergency ventilator designs can be tested and applied to rapidly enable access to ventilation.

## Supplementary Information

**Additional file 1.** Rabbit MV1 Carestation Flow Waveforms. Flow waveforms for rabbit MV1 during Carestation ventilation.

**Additional file 2.** Rabbit MV1 FALCON Flow Waveforms. Flow waveforms for rabbit MV1 during FALCON ventilation.

**Additional file 3.** Rabbit MV2 Carestation Flow Waveforms. Flow waveforms for rabbit MV2 during Carestation ventilation.

**Additional file 4.** Rabbit MV2 FALCON Flow Waveforms. Flow waveforms for rabbit MV2 during FALCON ventilation.

**Additional file 5.** Rabbit MV3 Carestation Flow Waveforms. Flow waveforms for rabbit MV3 during Carestation ventilation.

**Additional file 6.** Rabbit MV3 FALCON Flow Waveforms. Flow waveforms for rabbit MV3 during FALCON ventilation.

**Additional file 7.** Rabbit MV4 Carestation Flow Waveforms. Flow waveforms for rabbit MV4 during Carestation ventilation.

**Additional file 8.** Rabbit MV4 FALCON Flow Waveforms. Flow waveforms for rabbit MV4 during FALCON ventilation.

**Additional file 9.** Rabbit MV5 Carestation Flow Waveforms. Flow waveforms for rabbit MV5 during Carestation ventilation.

**Additional file 10.** Rabbit MV5 FALCON Flow Waveforms. Flow waveforms for rabbit MV5 during FALCON ventilation.

**Additional file 11.** Rabbit Pressure Waveforms. Pressure waveforms for rabbits MV1 to MV5 during Carestation and FALCON ventilation.

**Additional file 12.** Additional datasets. Additional datasets analyzed.

**Additional file 13.** Combine Pressures Python Script. Custom Python script used to combine pressure waveforms recorded from pressure sensors placed at the inspiratory and expiratory limbs during FALCON ventilation.

**Additional file 14.** Flow and Pressure Waveform Analysis Python Script. Custom Python script used to analyze flow and pressure waveforms.

## Data Availability

The flow waveform datasets (Additional files 1, 2, 3, 4, 5, 6, 7, 8, 9 and 10), pressure waveform datasets (Additional file 11), and other datasets (Additional file 12) analyzed, along with the custom Python code used to combine pressure waveforms (Additional file 13) and analyze the pressure and flow waveforms (Additional file 14) can be found in the supplementary material.

## Abbreviations

ABG: Arterial blood gas
ARDS: Acute respiratory distress syndrome
CEEV: Covid-19 era emergency ventilator
EtCO_2_: End-tidal carbon dioxide
I:E: Inspiratory time to expiratory time
MV: Mechanical ventilation
PaO_2_: Arterial partial pressure of oxygen
PaCO_2_: Arterial partial pressure of carbon dioxide
PEEP: Positive end expiratory pressure
PIP: Peak inspiratory pressure
RR: Respiratory rate
SB: Spontaneously breathing
SD1: Standard deviation 1 (short-term variation in Poincaré plot analysis)
SD2: Standard deviation 2 (long-term variation in Poincaré plot analysis)
SpO_2_: Blood oxygen saturation
V̇: Ventilation
V_T_: Tidal volume
